# Prepubertal Children With Metabolically Healthy Obesity or Overweight Are More Active Than Their Metabolically Unhealthy Peers Irrespective of Weight Status: GENOBOX Study

**DOI:** 10.3389/fnut.2022.821548

**Published:** 2022-04-12

**Authors:** Francisco Jesús Llorente-Cantarero, Rosaura Leis, Azahara I. Rupérez, Augusto Anguita-Ruiz, Rocío Vázquez-Cobela, Katherine Flores-Rojas, Esther M. González-Gil, Concepción M. Aguilera, Luis A. Moreno, Mercedes Gil-Campos, Gloria Bueno

**Affiliations:** ^1^Department of Specific Didactics, Faculty of Education, Institute of Biomedicine Research of Córdoba (IMIBIC), University of Córdoba, Córdoba, Spain; ^2^Center of Biomedical Research on Physiopathology of Obesity and Nutrition, Institute of Health Carlos III, Madrid, Spain; ^3^Unit of Investigation in Nutrition, Growth and Human Development of Galicia, Department of Pediatrics, University of Santiago de Compostela, Santiago de Compostela, Spain; ^4^Unit of Pediatric Gastroenterology, Hepatology and Nutrition, Department of Pediatrics, Instituto de Investigación Sanitaria de Santiago (IDIS), University Clinical Hospital of Santiago, Santiago de Compostela, Spain; ^5^Grupo de Nutrición, Alimentación, Crecimiento y Desarrollo (GENUD) Research Group, Instituto Agroalimentario de Aragón (IA2), Instituto de Investigación Sanitaria de Aragón (IIS Aragón), University of Zaragoza, Zaragoza, Spain; ^6^Department of Biochemistry and Molecular Biology II, Center of Biomedical Research, Institute of Nutrition and Food Technology “José Mataix”, University of Granada, Granada, Spain; ^7^Instituto de Investigación Biosanitariaibs, Granada, Spain; ^8^Metabolism and Investigation Unit, Maimónides Institute of Biomedicine Research of Córdoba (IMIBIC), Reina Sofia University Hospital, University of Córdoba, Córdoba, Spain; ^9^Unidad de Endocrinología Pediátrica, Hospital Clínico Lozano Blesa, Facultad de Medicina, Universidad de Zaragoza, Zaragoza, Spain

**Keywords:** physical activity, childhood, body mass index, metabolism, body composition, health, metabolic syndrome

## Abstract

**Background and Aim:**

The association of a metabolically healthy status with the practice of physical activity (PA) remains unclear. Sedentarism and low PA have been linked to increased cardiometabolic risk. The aim of this study was to evaluate the PA levels in metabolically healthy (MH) or unhealthy (MU) prepubertal children with or without overweight/obesity.

**Methods:**

A total 275 children (144 boys) with 9 ± 2 years old were selected for the GENOBOX study. PA times and intensities were evaluated by accelerometry, and anthropometry, blood pressure, and blood biochemical markers were analyzed. Children were considered to have normal weight or obesity, and further classified as MH or MU upon fulfillment of the considered metabolic criteria.

**Results:**

Classification resulted in 119 MH children (21% with overweight/obesity, referred to as MHO) and 156 MU children (47% with overweight/obesity, referred to as MUO). Regarding metabolic profile, MHO showed lower blood pressure levels, both systolic and diastolic and biochemical markers levels, such as glucose, Homeostatic Model Assessment of Insulin Resistance, triglycerides and higher HDL-c levels than MUO (*P* < 0.001). In addition, MHO children spent more time in PA of moderate intensity compared with MUO children. In relation to vigorous PA, MH normal weight (MHN) children showed higher levels than MUO children. Considering sex, boys spent more time engaged in moderate, vigorous, and moderate–vigorous (MV) PA than girls, and the number of boys in the MH group was also higher.

**Conclusion:**

Prepubertal MHO children are less sedentary, more active, and have better metabolic profiles than their MUO peers. However, all children, especially girls, should increase their PA engagement, both in terms of time and intensity because PA appears to be beneficial for metabolic health status itself.

## Introduction

Childhood obesity is a complex condition and can be associated with a number of cardiometabolic risk factors, including insulin resistance, dyslipidemia, and high blood pressure (1). To address this condition, the American Academy of Pediatrics recently recommended focusing on the clustering of metabolic and cardiovascular risk factors instead of defining metabolic syndrome (MetS) (2). The presence of comorbidities may vary depending on genetics, pubertal status, and ethnicity (3). Recently, criteria for the identification of children with obesity that are free from cardiometabolic risk, i.e., “metabolically healthy obesity”, have been defined (3). These other criteria are high-density lipoprotein cholesterol (HDL-c) > 40 mg/dL (or >1.03 mmol/L), triglycerides (TGs) ≤ 150 mg/dL (or ≤1.7 mmol/L), systolic and diastolic blood pressure (SBP and DBP) ≤ 90th percentile, and a fasting glycemia <100 mg/dL (3). However, the diagnosis of metabolic risk in prepubertal children is still compromised by the absence of reference cut-off points for specific sex, age, pubertal stage, and ethnicity groups. Moreover, metabolically healthy obesity status is not a stable condition because it can crossover to the “metabolically unhealthy obesity” phenotype during puberty (4). Therefore, it is important to consider the prepubertal stage to try to elucidate the mechanisms protecting against the appearance of a cluster of cardiometabolic risk factors (5).

Sedentary behaviors and physical activity (PA) seem to play a role in the metabolic phenotype (6). There is evidence that supports the positive effects of high levels of PA, low levels of sedentarism, and high levels of cardiorespiratory fitness (CRF) on cardiometabolic health (7), although there are discrepancies between published studies (8, 9). With respect to sedentary behaviors, most studies have not found any differences between metabolically healthy obesity and non-metabolically healthy obesity (8, 9), while PA and CRF are proposed to play a protective role against metabolically unhealthy obesity (10). However, there is a lack of studies assessing the association between PA and metabolically healthy obesity in children, despite the increasing number of lifestyle interventions aiming to achieve a healthier metabolic profile and a lower risk of cardiovascular mortality/morbidity (10). Providing new insights concerning the role of PA in metabolically healthy and unhealthy prepubertal children may be useful to avoid the onset of cardiometabolic comorbidities.

Therefore, the aim of the present study was to assess PA, including time and intensity, and sedentarism using accelerometers and to determine their association with metabolically healthy or unhealthy profile in a sample of Spanish prepubertal children with and without overweight/obesity.

## Materials and Methods

### Study Design

The present study was carried out in children recruited from three Spanish hospitals (Hospital Universitario Reina Sofía in Córdoba, Hospital Clínico Universitario in Santiago de Compostela, and Hospital Clínico Universitario Lozano Blesa in Zaragoza) under the framework of the GENOBOX study (11, 12). Participants were selected among those already attending these hospitals to confirm the diagnosis of overweight or obesity or for minor disorders that were not confirmed after clinical and laboratory investigations. For the current study, a subsample was selected using the following inclusion criteria: children >5 years of age and at the prepubertal stage (Tanner I), with the absence of endogenous obesity (caused by malfunction of the hormonal or metabolic system) or other metabolic or hormonal diseases, and with a minimum of valid accelerometer data (a minimum of 8 h of monitoring per day for at least 3 days, including at least 1 weekend day). Exclusion criteria were disease or malnutrition; the use of medications that alter blood pressure, glucose, or lipid metabolism; or not meeting the inclusion criteria.

Children and parents or guardians were informed about the purpose and procedures of the study and written consent was obtained from the parents/guardians, while children gave their assent to participate. The ethics committees of all of the participating institutions approved the study, which complied with the Declaration of Helsinki.

### Physical Examination

Medical history and physical examination were carried out, including the evaluation of sexual maturity and puberty classification according to Tanner's five-stage scale (13), which was validated by plasma sex hormone concentrations. Body weight, height, and waist circumference (WC) were measured following previously published protocols (14). The body mass index (BMI) z-score was calculated based on the Spanish reference standards published in (15).

Children with normal weight, overweight, and obesity were defined using the International Obesity Task Force (IOTF)'s age- and sex-specific BMI cut-off points that are equivalent to the adult values of 25 kg/m^2^ for overweight and 30 kg/m^2^ for obesity (16).

SBP and DBP measurements were taken in triplicate by the same examiner using an electronic manometer (Omrom, M6 AC) following international recommendations (17), and the mean of the two closest values was used in further analyses.

### Biochemical Analysis

Blood samples were drawn from the antecubital vein after an overnight fast. Routine blood tests were analyzed at the general laboratory of each participating hospital. Plasma glucose (CV = 1.0%), TGs (CV = 1.5%), total cholesterol (CV = 0.9%), HDL-c (CV = 0.8%), and low-density lipoprotein cholesterol (LDL-c) (CV = 1.5%) were measured using an automatic analyzer (Roche-Hitachi Modular P and D Autoanalyzer; Roche Laboratory Systems, Mannheim, Germany). Plasma insulin was analyzed by radioimmunoassay (CV = 2.6%) using an automatic microparticle analyzer (AxSYM; Abbott Laboratories, Abbott Park, IL, USA). Insulin resistance (IR) was assessed by means of the Homeostatic Model Assessment of IR (HOMA-IR) as insulin (μU/mL) × glucose (mmol/L)/22.5.

### Physical Activity Assessment by Accelerometry

ActiGraph GT3X+ accelerometers (ActiGraph; Pensacola, FL) were used to assess PA, and were programmed to collect raw acceleration data at a frequency of 30 Hz over a dynamic range time of 15 s (epochs). Accelerometers were placed over the right iliac crest and held in place using an adjustable elastic belt for 24 h a day to measure the accelerations of the segment where the monitor was connected. They were removed only for showering or nocturnal rest if the instrument caused discomfort during sleep. Parents and children were instructed that the child should wear the ActiGraph 24 h/day for 7 days. A minimum of 8 h of monitoring per day for at least 3 days, including at least 1 weekend day, was considered acceptable for the evaluation of PA and sedentary time (18).

Two rules were used to exclude low-quality data: all negative counts were replaced by missing data code and periods of 20 min or more of consecutive zero counts were replaced by missing data code prior to the analysis. Unavailability of valid data, non-compliance with the minimum number of hours set, or not enough time on valid days during the week or weekend were exclusion criteria for this analysis. The output generated by the ActiGraph GT3X+ included the total volume of PA and the cut-off points of PA intensity recommended by Evenson et al. (18) of sedentary: ≤100 counts per min (CPM); light: >100–2,296 CPM; moderate: 2,296–4,012 CPM; vigorous PA: ≥4,012 CPM.

### Metabolic Health Criteria

All children, independently of their BMI, where classified as metabolically healthy (MH) or unhealthy (MU) according to the published definition for metabolic syndrome in prepubertal children proposed by our group, based on previous authors' classifications (19, 20). This definition considers children as having MU status when fulfilling at least one of the following criteria: (1) SBP or DBP values higher or equal to the 90th percentile for age, sex, and height; (2) TG plasma concentrations higher than the 90th percentile for age, sex, and race; (3) HDL-c plasma concentration lower than the 10th percentile for age, sex, and race; (4) glucose plasma concentration higher or equal to 100 mg/dL; (5) HOMA-IR values higher than 2.5. Children that did not fulfill any of the mentioned criteria were considered to have an MH status. Finally, the sample was divided into the following four groups: metabolically healthy normal weight (MHN) group, metabolic unhealthy normal weight (MUN) group, metabolically healthy overweight/obesity (MHO) group, and metabolically unhealthy overweight/obesity (MUO) group.

### Statistical Analysis

The sample size estimation for the GENOBOX study was calculated based on the principal metabolic risk factors for cardiovascular disease associated with obesity. The calculation of the sample size was carried out for a 95% degree of confidence (type I error alpha = 0.05) and a power of 80% (beta error = 0.20) according to the estimation equation of *n* by comparison with two proportions of one variable in two independent groups. Under these conditions, the sample size was increased to a total of 300 to ensure that significant differences between children with obesity and normal weight could be found with a minimal difference of 20% for each examined parameter. All continuous variables were tested for normality using the Shapiro–Wilk and Kolmogorov-Smirnov tests, and the variables following a non-normal distribution were logarithmically transformed: SBP, HOMA-IR, TGs, HDL-c, and sedentary and vigorous PA. The homogeneity of variances was estimated using Levene's test. Differences between MH and MU children were analyzed by two-independent-sample *t*-tests or Mann–Whitney *U-*tests. χ^2^-tests were applied to categorical variables expressed as a percentage. Differences between the four groups based on MH/MU and weight status (MHN, MUN, MHO, and MUO) were analyzed using one-way ANOVA and Kruskal–Wallis test, and pairwise differences were assessed by *post-hoc* analyses, adjusted for age, to determine differences between experimental groups. Values in the descriptive tables and results are expressed as means and standard deviations. Differences were considered significant when *p* < 0.05. All statistical analyses were performed using IBM SPSS Statistics v.20 software.

## Results

A total of 275 children (144 boys) were included after excluding participants without valid accelerometer data or were missing information for metabolic risk and weight status or pubertal stage. Age, anthropometric measures, PA outcomes, and metabolically healthy and unhealthy status are shown in Table 1. The sample was homogeneously distributed in the MH and MU groups as well as by sex and age. Weight, BMI, and WC were higher in the MU group compared to the MH group, independently of age. Regarding moderate PA intensity, the MH group showed a higher engagement time (in minutes) than the MU group. MUN was the smallest group among both boys and girls.

**Table 1:** Demographic, anthropometric, and physical activity intensities compared between prepubertal children of metabolically healthy or unhealthy status.

	**MH group (*****N*** **=** **119)**		**MU group (*****N*** **=** **156)**		***p***
Age (years)	9.36 ± 1.87		8.91 ± 1.89		0.056
Weight (kg)	36.91 ± 10.71		45.77 ± 13.59		<0.001
Height (cm)	135 ± 10.22		137 ± 12.21		0.200
BMI (kg/m^2^)	20 ± 4.49		23.94 ± 4.58		<0.001
BMI z-score	0.84 ± 0.2		2.20 ± 0.18		<0.001
WC (cm)	69.6 ± 12.83		79.3 ± 12.58		<0.001
Sedentary (min/day)	458.38 ± 86.13		461.12 ± 91.53		0.203
LPA (min/day)	266.15 ± 49.73		270.81 ± 55		0.899
MPA (min/day)	39.52 ± 13.99		36.80 ± 13.32		0.045
VPA (min/day)	14.26 ± 8.59		13.01 ± 9.31		0.131
MVPA (min/day)	53.78 ± 20.89		49.80 ± 20.97		0.074
	***N***	**%**	***N***	**%**	***p^†^***
**Weight status**	119	43.5	156	56.5	<0.001
Normal	58	21.2	26	9.1	
Overweight/obesity	61	22.3	130	47.4	
**Sex**					0.464
Boys	67	24.1	77	28.5	
Girls	52	19.3	79	28.1	
**Boys**					<0.001
Normal weight	31	21.5	13	9	
Overweight	12	8.3	19	13.2	
Obesity	24	16.7	45	31.2	
**Girls**					<0.001
Normal weight	28	21.4	12	9.2	
Overweight	13	9.9	14	10.7	
Obesity	11	8.4	53	40.5	

*MH, metabolically healthy; MU, metabolic unhealthy; BMI, body max index; WC, waist circumference; LPA, low physical activity; MPA, moderate physical activity; VPA, vigorous physical activity; MVPA, moderate-to-vigorous physical activity*.

*Data are expressed as mean ± standard deviation. The p-values were obtained after two independent-samples t-tests or Mann–Whitney U-tests*.

† *The p-values were obtained after chi-squared tests*.

The variables included for the definition of metabolic syndrome by Olza et al. (19) were compared between the MHN, MUN, MHO, and MUO groups and are shown in Table 2. The subgroup of MHN children showed lower SBP and DBP than for both the MUN and MUO groups as well as a lower HOMA-IR than the MUO group. We further observed a higher HDL-c in the MHN group (*p* < 0.001) than in the other groups, with the latter showing a linear decrease from MHN to MUO. Finally, the MHO group presented lower TG levels than the MU subgroups. Although differences were found between the MH and MU groups in relation to glucose, none of the values were considered pathological.

**Table 2:** Measurements of metabolic risk markers in prepubertal children according to metabolically healthy or unhealthy status and normal or overweight/obesity.

**Metabolic syndrome variables**	**MHN group (*N* = 59)**	**MUN group (*N* = 25)**	**MHO group (*N* = 60)**	**MUO group (*N* = 131)**	***p***
SBP (mm Hg)	99 ± 7.5^a^	106 ± 10.5^b^	102 ± 8.8^a^^b^	113 ± 12.2^c^	<0.001
DBP (mm Hg)	59 ± 6.5^a^	64 ± 10.4^a^^b^	61 ± 6.7^a^	68 ± 10.3^b^	<0.001
Glucose (mg/dL)	83 ± 6.5^a^	86 ± 8.2^b^	81 ± 6.6^a^	84 ± 8.6^a^^b^	0.003
HOMA-IR	1.1 ± 0.5^a^	1.7 ± 1.0^a^	1.3 ± 0.6^a^	2.6 ± 1.6^b^	<0.001
TG (mg/dL)	52 ± 15.1^a^	72 ± 35.1^b^	50 ± 17.7^a^	74 ± 31.5^b^	<0.001
HDL-c (mg/dL)	66 ± 13.4^c^	58 ± 15.3^a^	53 ± 12.4^a^^b^	47 ± 12.4^b^	<0.001

*MHN, metabolically healthy normal weight group; MUN, metabolic unhealthy normal weight group; MHO, metabolically healthy overweight/obesity group; MUO, metabolic unhealthy overweight/obesity group; SBP, systolic blood pressure; DBP, diastolic blood pressure; HOMA-IR, Homeostatic Model Assessment of Insulin Resistance; TG, triglycerides*.

*Data are expressed as mean ± standard deviation. The p-values were obtained by two-way ANOVA or Kruskal–Wallis test. Non-matching superscript letters (^a^, ^b^, or ^c^) indicate significant differences (p < 0.05) by pairwise post-hoc tests adjusted for age to determine differences between experimental groups*.

**Figure 1:**
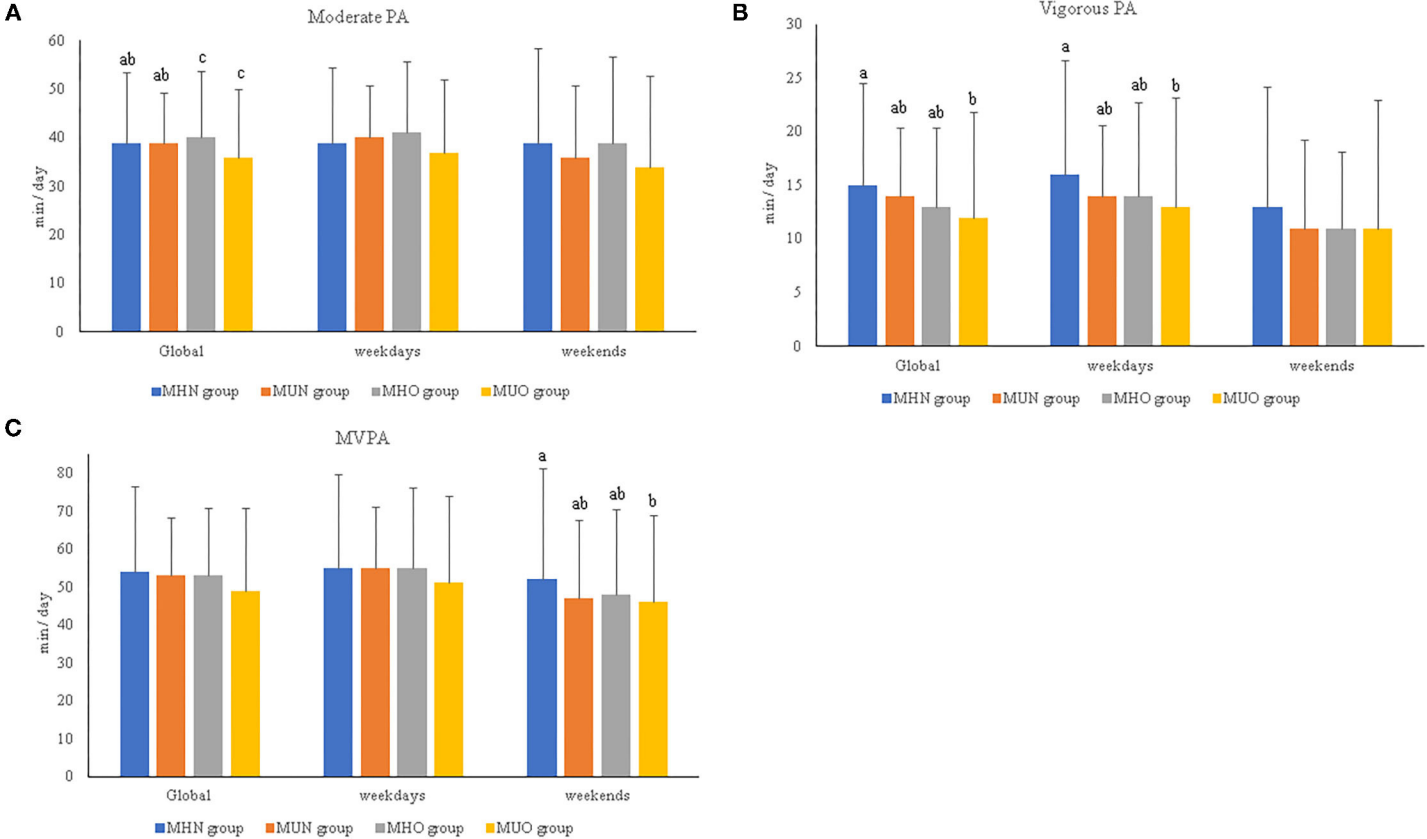
**(A–C)** Relevant results of physical activity intensities and duration per day in prepubertal children according to metabolically healthy or unhealthy status and normal weight or overweight/obesity. MHN, metabolically healthy normal weight group; MUN, metabolic unhealthy normal weight group; MHO, metabolically healthy overweight/obesity group; MUO, metabolic unhealthy overweight/obesity group; PA, physical activity; MVPA, moderate-to-vigorous physical activity. The *p-*values were obtained two-way ANOVA or Kruskal–Wallis test. Non-matching superscript letters (^a^, ^b^, or ^c^) indicate significant differences (*p* < 0.05) by pairwise *post-hoc* tests adjusted for age and sex to determine differences between experimental groups. The data are subdivided in global data, by weekend, or by weekdays.

After adjusting for age, the results show that the MUO children spent less time (in min) engaged in moderate-intensity PA than the MHO children (*p* = 0.039), as well as less time (in min) engaged in vigorous-intensity PA than the MHN children (*p* = 0.032; Figure 1).

By contrast, when children with obesity were studied separately (i.e., without including overweight and normal weight) (Supplementary Table 1), the MH children with obesity spent more time engaged in moderate-intensity PA (min) (41.76 ± 11.85) than the MU children with obesity (36.42 ± 14.20; *p* = 0.034). In addition, the MHN children spent more time engaged in vigorous-intensity PA (15.42 ± 9.54) than the MU children with obesity (13.19 ± 10.27; *p* = 0.050). In relation to PA practice during the week and on weekends, the MHN children showed more time engaged in vigorous-intensity PA during the weekdays as well as moderate–vigorous PA (MVPA) during the weekend compared with the MUO children (*p* < 0.05; Figure 1).

**Figure 2:**
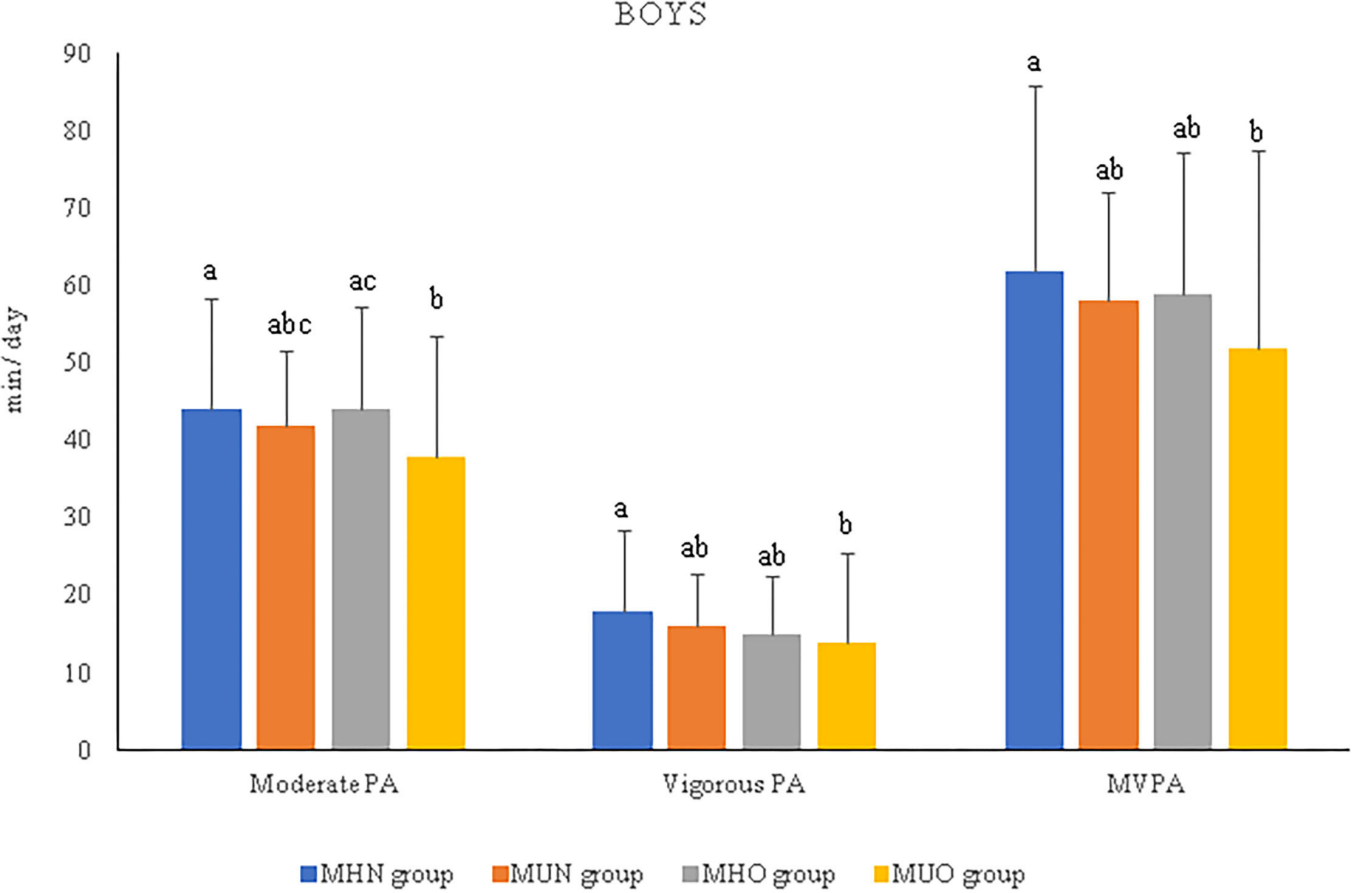
Relevant results of physical activity intensities and duration per day in prepubertal boys according to metabolically healthy or unhealthy status, normal weight or overweight/obesity. MHN, metabolically healthy normal weight group; MUN, metabolic unhealthy normal weight group; MHO, metabolically healthy overweight/obesity group; MUO, metabolic unhealthy overweight/obesity group; PA, physical activity; MVPA, moderate-to-vigorous physical activity. The *p-*values were obtained two-way ANOVA or Kruskal–Wallis test. Non-matching superscript letters (^a^, ^b^, or ^c^) indicate significant differences (*p* < 0.05) by pairwise *post-hoc* tests adjusted for age to determine differences between experimental groups.

After stratifying the analyses by sex, the results showed that boys engaged in more moderate PA than girls (41.23 ± 14.93 vs. 34.92 ± 11.73), vigorous PA (15.40 ± 10.58 vs. 11.96 ± 7.1), and MVPA (56.31 ± 22.49 vs. 46.91 ± 17.29) in the total sample (*p* < 0.005), as well as within MH/MU groups (Supplementary Table 2). Similar results were found for both the MHN and MHO groups. Only for MHN children was light PA also higher in boys than in girls (*p* < 0.05). When sex was studied independently, the MUO boys showed a lower time engaged in moderate-intensity PA (*p* = 0.019) than the MHO boys, as well as in vigorous-intensity PA and MVPA (*p* = 0.032 and *p* = 0.031, respectively) than the MHN boys (Figure 2). No differences were found between the groups of girls (Supplementary Table 2).

## Discussion

In the present study, MH children, even with obesity, were shown to spend more time engaged in objectively measured PA than children with at least one metabolic risk factor (i.e., the MU group). In the literature, there are inconsistent findings due to the information bias associated with MVPA and sedentary behavior being measured using questionnaires or pedometers (9, 21), which lack intensity estimates. Moreover, some previous studies have examined the association between PA and sedentary time and metabolic risk markers, but only a few have categorized children and adolescents as metabolically healthy/unhealthy across weight status categories (22, 23), and most lacked accelerometry measurements. The different protocols or methods used, as well as the other factors such as age, sex, or puberty, could have influenced the changes in metabolic status.

The homogeneous sample recruited by sex and age, considering only prepubertal children but with overweight and obese children represented, is a strength of the current study. Indeed, puberty can be a confounding factor since it is a crucial period for insulin resistance and the development of metabolic syndrome (4). An estimate of gluteofemoral fat mass, has now been established as an important determinant of MH in adults, independently of subcutaneous abdominal fat mass and visceral fat mass (24). One limitation in this study is the classification according to weight using BMI, since it was not possible to obtain data to estimate adiposity in all the children, nor the use of other techniques such as DXA. So, BMI cut off were used similarly as in the clinical practice. For pediatric population, especially in prepuberal, at this moment there are not waist or hip circumference reference values but it is known that body composition is modulated at puberty (4). There is an increase in fat mass in pubertal girls and alongside greater lean mass, the adolescent boys frequently exhibit less total fat mass but similar (or greater in some cases) central fat mass than do girls. In Genobox sample as published by our group, there are no relevant differences in total fat and lean mass between sex (25). Regardless of sex and its influence, an adequate cardiorespiratory fitness in childhood and adolescence has been associated with decreased fat mass over time (26) although is not strongly associated with MHO phenotype (21). So, different intensities and frequency of physical activity seems to influence a healthy fitness condition.

In the same way, there are not always age- or sex-specific reference values for some of these biochemical parameters in children, but there are differences in cardiometabolic risk factors and metabolic syndrome diagnosis along the pubertal stages that must be considered when evaluating the future risk and implicated factors (4, 27). In the present study, 47.4% of the children with overweight or obesity were MU, as well as 9% of the children of normal weight, compared to other studies also carried out in children (23, 28). The reason for these differences could be that cardiometabolic risk is very precocious in relation to age and prepubertal stage, as well as to the classification used to define MU status. Thus, future longitudinal research during childhood is needed to determine the true risk and cut-offs that are at least age- and sex-adjusted.

We also selected specific criteria (19) to identify prepubertal MUO children based on the highest percentile values for BP, TGs, and HDL-c for minimum age and sex, glucose plasma concentration higher or equal to 100 mg/dL, or HOMA-IR values higher than 2.5, which were used as the cut-offs for the prepubertal children in the present study and were based on what other authors have previously established (29).

Childhood obesity has been associated with a moderately increased risk of adult obesity-related morbidity, but BMI is not a good predictor of the incidence of these morbidities. Often, it occurs in adults who were of a healthy weight in childhood. Therefore, targeting obesity reduction solely in children with overweight or obesity may not substantially reduce the overall burden of obesity-related diseases in adulthood (30). In the IDEFICS study (Identification and prevention of Dietary- and lifestyle-induced health EFfects In Children and infantS) performed with preadolescents, physical inactivity and sedentary lifestyle were also found to be associated with the development of insulin resistance, independently of weight status (31). In fact, in our study, MHN children had a better metabolic profile with lower SBP and HOMA-IR values and higher HDL-c compared with MUO children, also showing lower serum concentrations of glucose and triglycerides. Thus, perhaps we should also focus on other metabolic risk factors associated with low PA practice, and not only on obesity status. In a review by Kuzik et al. (23), 20 studies with 4,581 children and adolescents were included, and it was reported that each additional 60 min of sedentary time per day was associated with 8–11% higher odds of being classified as MU in the normal weight group, compared to being classified as MH. However, each additional 10 min of MVPA per day was associated with lower odds of MU classification in both the normal weight and overweight groups, as compared to being classified as MH. These results are complementary to ours, showing the principal role of time and intensity of PA in determining the status of metabolic health.

PA has been suggested to modulate fuel metabolism. The increased fat oxidation promoted by PA might be the basis for the prevention and restoration of insulin sensitivity and reduction of metabolic syndrome in children with obesity. Primarily, vigorous PA can decrease energy stores, improve body composition (by increasing lean mass as a substitute of fat mass lost), and restore fat distribution (by reducing visceral and intramuscular fat depots) (32). In another study, time spent engaging in PA for most children was lower than the 60 min of MVPA per day recommended by the WHO (33), being more alarming in girls, in which the time spent was even lower. In that study, MHO children spent more time engaged in moderate-intensity PA than the MUO children. In relation to vigorous activity, the MHN children showed higher levels than the MUO children. It is known that metabolic benefits are greater with MVPA practice than for lower-intensity PA, and decreasing sedentary time seems to be beneficial only for metabolic health (23). In the IDEFICS study, MVPA at baseline (upper two quartiles) showed a protective effect on the development of insulin resistance 2 years later also for children with normal weight at baseline, which indicates that the negative effect of low PA is not just mediated by obesity (31). In a study by Stabelini et al. (34), time spent engaging in MVPA was inversely associated with a continuous metabolic syndrome risk score (31) in both sexes. In most of the studies that have considered sex, boys display higher MVPA levels compared to girls (35, 36), as can be observed in our study, though in the MH subgroup of children with normal weight and obesity. In addition, MVPA time has been reported to be significantly higher in children on weekdays compared to at weekends (35). In the present study, the MHN children spent more time overall and also in greater intensity PA, i.e., in vigorous PA and MVPA, during the week and at the weekend compared with MUO children.

Although sedentary time did not indicate significant differences, it seems that the MHO children were less sedentary than the MHN and MUO children. In other recent article of our group on this subsample of children, a higher percentage of active children were reported to be members of a sport club or practiced collective sports when compared to sedentary children (12). The MHO children in this study, selected from hospitals, likely practice PA to control their weight and to be healthier. It has also been described that a decrease in MVPA and an increase in sedentary time after follow-up are significantly lower for children who participate in sports than for those who do not, both for boys and girls (36). Some authors have associated overweight with low levels of PA and sedentary behavior (37), but it seems that there is no association between sedentary time and metabolic health in people with obesity (8, 23). In a recent systematic review of 26 studies in children and adolescents, the MVPA levels in children with or without obesity were consistently below the recommendations, without marked differences in sedentary time between children with obesity and normal weight (38).

Engaging in PA during childhood can induce biomechanical, physiological, and psychological changes, resulting in beneficial adaptations that persist throughout adulthood (34). Therefore, it is important to identify the protective factors, avoiding the crossover of the MHO (considering adiposity) to MU phenotype. The principal limitation in this study is that there is no consensus about what traditional metabolic features use in children and how to estimate adiposity instead of using variables as BMIzscore. Metabolically unhealthy might be confounding especially when accompanied by obesity category. Research about metabolic health and its relationship with weight status and other factors related to PA should be included in individualized clinical interventions in children by determining specific lifestyle modifications providing the most health benefit for them. In conclusion, MHO children are more active than their MUO peers, and they have a better metabolic profile, so this active condition appears to be beneficial for metabolic health itself. However, all children, especially girls, should increase their PA engagement, both in terms of time and intensity.

## Data Availability Statement

The raw data supporting the conclusions of this article will be made available by the authors, without undue reservation.

## Ethics Statement

The studies involving human participants were reviewed and approved by Ethics Committees from Hospital Universitario Reina Sofía in Córdoba, Hospital Clínico Universitario in Santiago de Compostela, and Hospital Clínico Universitario Lozano Blesa in Zaragoza (Spain). Written informed consent to participate in this study was provided by the participants' legal guardian/next of kin.

## Author Contributions

FL-C: conceptualization, data curation, formal analysis, methodology, and writing—original draft. RL: conceptualization, funding acquisition, investigation, methodology, project administration, resources, and writing—review and editing. AR: data curation, investigation, resources, and writing—review and editing. AA-R: data curation, formal analysis, software, and writing—review and editing. RV-C, KF-R, and EG-G: data curation, resources, and writing—review and editing. CA and LM: conceptualization, funding acquisition, investigation, project administration, and writing—review and editing. GB: conceptualization, funding acquisition, investigation, project administration, resources, and writing—review and editing. MG-C: conceptualization, investigation, methodology, project administration, resources, and writing—original draft. All authors contributed to the article and approved the submitted version.

## Funding

This work was supported by the Plan Nacional de Investigación Científica, Desarrollo e Innovación Tecnológica (I + D + I), Instituto de Salud Carlos III-Health Research Funding (FONDOS FEDER) (PI051968, PI11/02042, PI11/02059, PI11/01425 and PI16/00871, PI16/01301, PI16/01205), Redes temáticas de investigación cooperativa RETIC (Red SAMID RD12/0026/0015), and the Mapfre Foundation. The authors also acknowledge Instituto de Salud Carlos III for personal funding: Contratos i-PFIS: doctorados IIS-empresa en ciencias y tecnologías de la salud de la convocatoria 2017 de la Acción Estratégica en Salud 2013–2016 (IFI17/00048) and the Spanish Ministry of Education (FPU 16/03653).

## Conflict of Interest

The authors declare that the research was conducted in the absence of any commercial or financial relationships that could be construed as a potential conflict of interest. The handling editor JD-C declared a shared affiliation “University of Granada” with the authors AA-R, EG-G, and CA at the time of review.

## Publisher's Note

All claims expressed in this article are solely those of the authors and do not necessarily represent those of their affiliated organizations, or those of the publisher, the editors and the reviewers. Any product that may be evaluated in this article, or claim that may be made by its manufacturer, is not guaranteed or endorsed by the publisher.
