# Supplementary material for: Prepubertal Children With Metabolically Healthy Obesity or Overweight Are More Active Than Their Metabolically Unhealthy Peers Irrespective of Weight Status: GENOBOX Study
Source: Front Nutr. 2022 Apr 12;9:821548. doi: 10.3389/fnut.2022.821548 (PMC9040553; doi:10.3389/fnut.2022.821548)
Supplement: Supplementary file 1 [file Table_1.docx]

Supplementary table 1. Physical activity intensities and duration per day in prepubertal children according to metabolically healthy or unhealthy status and normal weight or overweight/obesity.

|  | MHN group  (59) | MUN group  (25) | MHO group  (60) | MUO group  (131) | *p* |
| --- | --- | --- | --- | --- | --- |
| Sedentary (S) (min/day) | 466 ± 80.6 | 461 ± 84.1 | 451 ± 91.3 | 461 ± 93.0 | 0.737 |
| S weekdays | 472 ± 87.0 | 459 ± 73.5 | 459 ± 93.8 | 461 ± 89.2 | 0.985 |
| S weekends | 451 ± 100.0 | 465 ± 126.7 | 431 ± 116.6 | 457 ± 126.3 | 0.260 |
| Light (L) PA (min/day) | 260 ± 50.7 | 286 ± 42.7 | 272 ± 48.5 | 265 ± 61.3 | 0.582 |
| L weekdays | 254 ± 49.4 | 284 ± 45.0 | 271 ± 53.3 | 265 ± 64.6 | 0.063 |
| L weekends | 274 ± 68.1 | 292 ± 49.5 | 275 ± 6.0 | 270 ± 74.1 | 0.570 |
| Moderate (M) PA (min/day) | 39 ± 14.4 ^ab^ | 39 ± 10.3 ^ab^ | 40 ± 13.7 ^b^ | 36 ± 14.0 ^c^ | 0.235 |
| M weekdays | 39 ± 15.4 | 40 ± 10.8 | 41 ± 14.8 | 37 ± 14.9 | 0.207 |
| M weekends | 39 ± 19.4 | 36 ± 14.7 | 39 ± 17.7 | 34 ± 18.7 | 0.151 |
| Vigorous (V) PA (min/day) | 15 ± 9.5 ^a^ | 14 ± 6.4 ^ab^ | 13 ± 7.4 ^ab^ | 12 ± 9.8 ^b^ | 0.191 |
| V weekdays | 16 ± 10.7 ^a^ | 14 ± 6.6 ^ab^ | 14 ± 8.7 ^ab^ | 13 ± 10.2 ^b^ | 0.024 |
| V weekends | 13 ± 11.2 | 11 ± 8.2 | 11 ± 7.1 | 11 ± 12.0 | 0.175 |
| MVPA (min/day) | 54 ± 22.6 | 53 ± 15.2 | 53 ± 17.8 | 49 ± 21.9 | 0.323 |
| MVPA weekdays | 55 ± 24.7 | 55 ± 16.0 | 55 ± 21.2 | 51 ± 23.0 | 0.214 |
| MVPA weekends | 52 ± 29.3 ^a^ | 47 ± 20.5 ^ab^ | 48 ± 22.6 ^ab^ | 46 ± 23 ^b^ | 0.028 |

MHN, metabolically healthy normal weight group; MUN, metabolic unhealthy normal weight group; MHO, metabolically healthy overweight/obesity group; MUO, metabolic unhealthy overweight/obesity group; PA, physical activity; MVPA, moderate-to-vigorous physical activity.

Data are expressed as mean ± standard deviation. The *p-*values were obtained two-way ANOVA or Kruskal–Wallis test. Non-matching superscript letters (^a^, ^b^, or ^c^) indicate significant differences (*p* < 0.05) by pairwise post hoc tests adjusted for age to determine differences between experimental groups.

Supplementary table 2. Physical activity intensities and duration per day in prepubertal children according to metabolically healthy or unhealthy status, normal weight or overweight/obesity, and sex.

|  | Boys | | | | | Girls | | | | |
| --- | --- | --- | --- | --- | --- | --- | --- | --- | --- | --- |
|  | MHN group  (31) | MUN group  (13) | MHO group  (36) | MUO group  (64) | *p* | MHN group  (28) | MUN group  (12) | MHO group  (24) | MUO group  (67) | *p* |
| Sedentary time(min/day) | 455 ± 85.9 | 474 ± 104.0 | 457 ± 110.4 | 457 ± 99.2 | 0.822 | 479 ± 73.7 | 446 ± 56.5 | 442 ± 51.98 | 465 ± 87.7 | 0.492 |
| Light PA (min/day) | 264 ± 49.2 | 287 ± 45.9 | 277 ± 49.4 | 264 ± 57.1 | 0.507 | 256 ± 52.9 | 285 ± 40.9 | 264 ± 47.1 | 271 ± 56.6 | 0.923 |
| Moderate PA (min/day) | 44 ± 14.5 ^a^ | 42 ± 9.7 ^abc^ | 44 ± 13.2 ^ac^ | 38 ± 15.6 ^b^ | 0.050 | 33 ± 12.1 | 36 ± 10.6 | 35 ± 12.7 | 35 ± 11.9 | 0.998 |
| Vigorous PA (min/day) | 18 ± 10.6 ^a^ | 16 ± 6.7 ^ab^ | 15 ± 7.5 ^ab^ | 14 ± 11.6 ^b^ | 0.163 | 13 ± 7.4 | 11 ± 5.0 | 11 ± 6.8 | 12 ± 7.6 | 0.712 |
| MVPA (min/day) | 62 ± 23.9 ^a^ | 58 ± 14.1 ^ab^ | 59 ± 18.1 ^ab^ | 52 ± 25.4 ^b^ | 0.112 | 46 ± 18.0 | 47 ± 15.0 | 46 ± 18.5 | 47 ± 17.9 | 0.987 |

MHN, metabolically healthy normal weight group; MUN, metabolic unhealthy normal weight group; MHO, metabolically healthy overweight/obesity group; MUO, metabolic unhealthy overweight/obesity group; PA, physical activity; MVPA, moderate-to-vigorous physical activity.

Data are expressed as mean ± standard deviation. The *p-*values were obtained two-way ANOVA or Kruskal–Wallis test. Non-matching superscript letters (^a^, ^b^, or ^c^) indicate significant differences (*p* < 0.05) by pairwise post hoc tests adjusted for age to determine differences between experimental groups.
